# Biosynthesis and Characterization of Silver Nanoparticles Produced by *Parachlorella kessleri* and *Cyclotella* spp., and the Evaluation of Their Antibacterial Activity

**DOI:** 10.3390/ijms241310599

**Published:** 2023-06-25

**Authors:** Khadija El Ouardy, Rkia Lbouhmadi, Hind Attaoui, Mustapha Mouzaki, Hanane Mouine, Zohra Lemkhente, Youssef Mir

**Affiliations:** Faculty of Medicine and Pharmacy of Agadir, University Ibn Zohr, Agadir 80060, Morocco

**Keywords:** biosynthesis, microalgae, silver nanoparticles, antibacterial

## Abstract

Green synthesis is one of the fastest and best ways for ecofriendly nanoparticle synthesis. This study aims to investigate the use of the green microalgae *Parachlorella kesseleri* and *Cyclotella* spp. for the biological synthesis of silver nanoparticles (AgNPs). This work focuses on optimizing various parameters necessary for the production and stability of AgNPs. The nanoparticle formation was confirmed by UV-Visible analysis, which revealed the surface plasmon resonance band at 420 nm. The characterization of the AgNPs was performed using UV-visible spectroscopy, X-ray diffraction (XRD), scanning electron microscopy coupled with energy dispersive X-ray microanalysis (SEM-EDS), Fourier transform infrared spectroscopy (FTIR), and inductively coupled plasma atomic emission spectroscopy (ICP-AES). The antimicrobial properties of these bioactive AgNPs were also tested, showing excellent antibacterial activity against six bacterial strains, *Escherichia coli*, multidrug-resistant *Escherichia coli*, *Bacillus clausii*, *Pseudomonas aeruginosa*, *Staphylococcus aureus*, and *Salmonella typhi*. The biosynthesis of AgNPs from living cultures of microalgae has remarkable antibacterial properties. Other studies are underway in our laboratory to clarify the mechanism of the biosynthesis of these nanoparticles, and their action on bacteria.

## 1. Introduction

The nanotechnologies represent a remarkable advance for our time, offering practical solutions that simplify our daily lives. Nanoscience and nanotechnology involve the creation of new materials, tools and processes by manipulating atoms on a scale of 1 to 100 nm. This field has made significant contributions to research in a variety of disciplines, including agriculture, materials science, health, engineering, computing, biology and chemistry [1,2,3]. In particular, when metals are reduced to nanometric dimensions, they exhibit physical, chemical and biological properties that differ from those of their larger counterparts. In recent years, numerous studies have focused on the characteristics and applications of nanoparticles. The unique properties of metallic nanoparticles have attracted the attention of scientists for various applications [4,5].

Among the different types of metallic nanoparticles, those composed of gold, platinum and silver have attracted a great deal of interest for their potential therapeutic applications. These nanoparticles have demonstrated remarkable therapeutic activities in a variety of medical conditions, including tumors, diabetes, infectious diseases, and many others [6,7].

Recent publications have shown that silver nanoparticles (AgNPs) possess the potential to serve as antibacterial agents [8,9]. However, the functional properties of AgNPs can be enhanced by using ecologically friendly synthesis methods. The utilization of green methods for the biosynthesis of AgNPs is becoming increasingly popular for its sustainable nature compared to other methods. Physical and chemical methods, like ion spraying and chemical reduction, have higher energy requirements and lower economic feasibility [10]. Eco-friendly synthesis methods, such as those utilizing plants, algae, and bacteria, are considered to be more reproducible and stable. Additionally, microalgae-mediated synthesis is considered the fastest and least complex of these methods [11,12,13].

Despite the many studies that have been conducted on microalgae and their ability to produce nanoparticles, there is still a need for further research to fully understand the diverse potential of these microorganisms and to optimize their use as a platform for producing nanoparticles. The diversity and chemical complexity of microalgal species highlights the importance of ongoing exploration in this field.

*Parachlorella kessleri* and *Cyclotella* spp. are two green microalgae known for their small size, fast growth rate, and ability to survive in various environmental conditions. These microalgae have been broadly studied for their inherent use in many domains, such as biotechnology, bioremediation, and biofuel production. Additionally, dried biomass and extracts of *P. kessleri* were utilized as a green biosynthetic agent in the production of AgNPs [14,15]. To our knowledge, the ability to biosynthesize AgNPs has not yet been realized using living cells of *Cyclotella* spp. nor *P. kessleri*.

The present work describes a new green technic to synthetize metallic nanoparticles using precursors consisting of living cells of microalgae *Cyclotella* spp. and of *P. kessleri*. Various physic-chemical techniques were used to characterize those AgNPs such as SEM-EDS, XRD, FTIR. The evaluation of their antibacterial activity was performed using four Gram-negative bacteria: multidrug-resistant *Escherichia coli*, *E. coli*, *Pseudomonas aeruginosa*, and *Salmonella typhi,* and two Gram-positive bacteria: *Bacillus clausii* and *Staphylococcus aureus.*

## 2. Results and Discussion

In the current research, *P. kesseleri* and *Cyclotella* spp. microalgae were utilized as bioreactors to reduce silver ions to AgNPs through the utilization of phytochemicals found in the microalgae. The progression of the reaction was monitored utilizing spectroscopic analysis.

### 2.1. UV-Vis Spectroscopy of AgNPs

**Figure 1:**
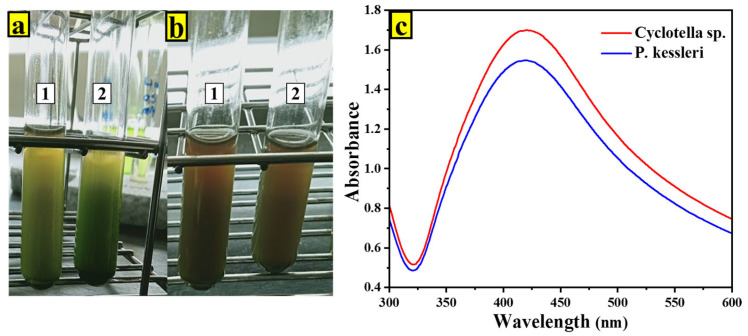
Color change before (**a**) and after AgNP formation (**b**) using microalgae as a precursor and 1 mM of AgNO_3_, (Tube (1) *P. kesseleri*, tube (2) *Cyclotella* spp.); and (**c**) absorption curve of AgNPs formed using 1 mM of AgNO_3_, and exposed to light for 24 h at room temperature.

After a few hours, the color was observed to change from green to brown. This change is caused by the excitation of the vibrational bands linked to the surface plasmon resonance, which reveals the transformation of Ag+ ions into Ag0 and the generation of AgNPs (Figure 1a,b). A surface plasmon resonance peak was found around 430 nm using UV-Vis spectroscopy, which confirms previous visual observations (Figure 1c).

The UV-visible analysis revealed that both microalgae strains, *P. kesseleri* and *Cyclotella* spp., successfully reduced the metal precursor to form metal nanoparticles, exhibiting a prominent peak at approximately 430 nm. The observed slight differences in the UV-visible peaks of the synthesized AgNPs using these two microalgal strains can be attributed to various factors. These factors may include the presence of distinct biomolecules on the surface of the AgNPs and variations in the synthesis mechanisms employed by the microalgae strains. Notably, previous studies have reported variations in the surface plasmon resonance (SPR) characteristics of silver nanoparticles synthesized by different species, leading to SPR peaks at different wavelengths within the range of 410 nm to 460 nm [16,17].

**Figure 2:**
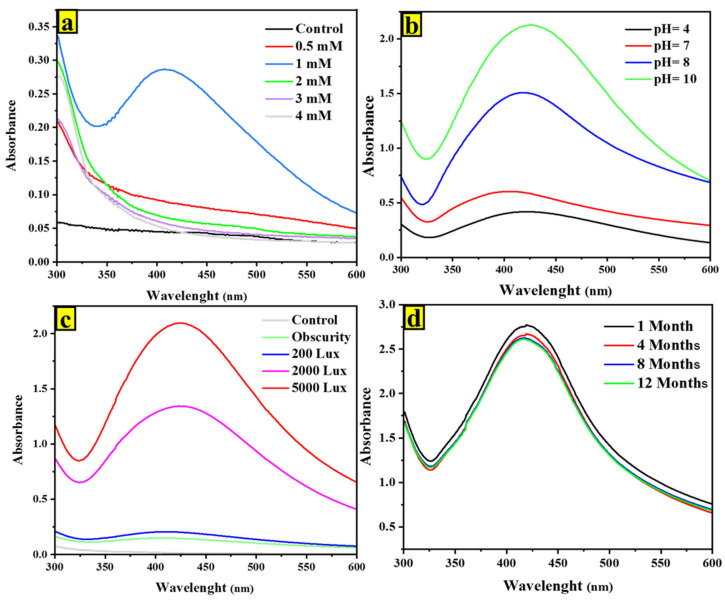
UV-visible spectrum of factors optimized for nanoparticle synthesis by *P. kesseleri* after 24 h: (**a**) at different AgNO_3_ concentrations under light; (**b**) at different pH under light and using 1 mM of AgNO_3_; and (**c**) at different light intensities using 1 mM of AgNO_3_. (**d**) Stability curve.

**Figure 3:**
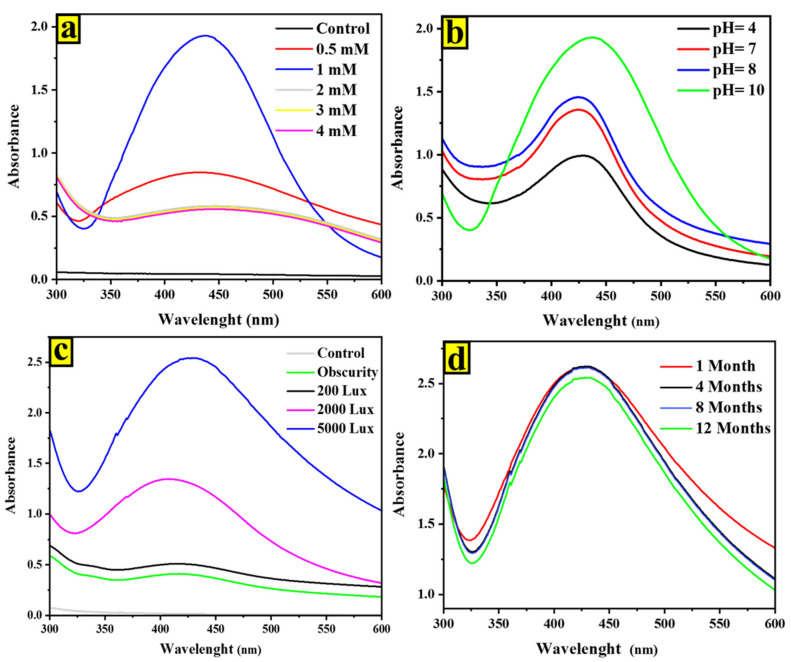
UV-visible spectrum of factors optimized for nanoparticle synthesis by *Cyclotella* spp. after 24 h: (**a**) at different AgNO_3_ concentrations under light; (**b**) at different pH under light and using 1 mM of AgNO_3_; and (**c**) at different light intensities using 1 mM of AgNO_3_. (**d**) Stability curve.

In this work, the biosynthesis process for nanoparticles was optimized, and we found that 1 mM of AgNO_3_ is the optimal concentration for the metal precursor (Figure 2a and Figure 3a). As the AgNO_3_ concentration increased (0.5, 1, 2, 3, 4 mM), the intensity of the silver nanoparticle absorption peak became more intense, while the wavelength of the peak remained constant. Notably, the highest peak intensity was observed at an AgNO_3_ concentration of 1 mM. The decrease in absorption peak intensity at higher AgNO_3_ concentrations could be attributed to the formation of an AgNO_3_ layer around the growing particles [18,19].

It was noted that pH and light are crucial factors in the biosynthesis of AgNPs, particularly in regard to the rate of reduction. In this case, increasing light intensity and using an alkaline pH improved the formation of AgNPs (Figure 2 and Figure 3), which aligns with the findings of other investigations [20,21]. Chitra et al. previously noted that at alkaline pH, the interactions between the positive charge of silver ions and the negative charge of functional groups are amplified. Additionally, the conclusions of Yurtluk et al. supported the findings of this work as well, which reported a better synthesis at pH 10 compared to pH values below 10 using *Bacillus* sp. In all pH solutions, both microalgae strains exhibited distinct absorption spectra of AgNPs (Figure 2b and Figure 3b). However, at lower pH levels, a peak shift towards 450 nm was observed compared to pH 10, which exhibited a narrower spectrum with a maximum around 420 nm. This finding aligns with a study conducted by Godínez et al. [22], who also observed a similar trend in AgNPs generated with Spirulina platensis at neutral and basic pH levels. The impact of light intensity on the biosynthesis of AgNPs by microalgae is significant (Figure 2c and Figure 3c). Our results suggest that microalgal cells have the potential to produce AgNPs when exposed to light, but no AgNPs are detected in the dark, which is consistent with previous research [23,24]. The spectra of the AgNPs produced showed a λmax value around 420 nm, and the peak intensity increased with an increase in light intensity. This indicates that the rate of conversion from Ag^+^ to Ag^0^ can be effectively enhanced by visible-light exposure.

The stability of the AgNPs was evaluated over a twelve-month period at room temperature by monitoring the SPR peaks. The results showed that even after twelve months, there were no observable changes in the optical characteristics of the nanoparticle solution, indicating that the nanoparticles are highly stable (Figure 2d and Figure 3d). This stability may be due to the presence of stabilizing agents within the microalgal cells [25,26].

### 2.2. Characterization of AgNPs

**Figure 4:**
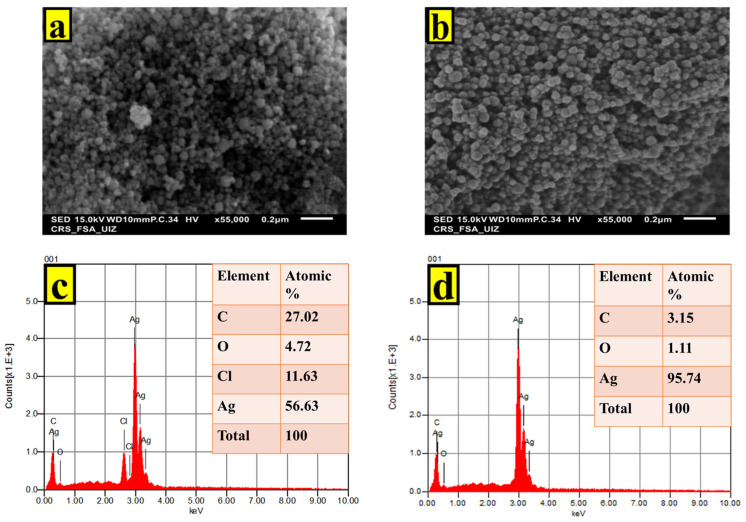
SEM micrograph of (**a**) *P. kesseleri* AgNPs and (**b**) of *Cyclotella* spp AgNPs. EDS results indicating sharp peak for (**c**) *P. kesseleri* AgNPs and (**d**) for *Cyclotella* spp. AgNPs.

SEM images and energy dispersive spectrometry (EDS) data confirmed the successful synthesis of AgNPs using both microalgae strains (Figure 4). The SEM images revealed the presence of spherical nanoformulations, with sizes ranging from 20 to 50 nm for both microalgae strains. Additionally, the EDS analysis exhibited a distinct absorption peak at 3 keV, indicating the presence of Ag in the synthesized nanoparticles.

Further analysis of the EDS spectra provided valuable insights into the elemental composition of the AgNPs. In the case of *P. kesseleri* AgNPs, the spectral analysis clearly showed the presence of oxygen, chlorine, and carbon elements. Conversely, the EDS analysis of *Cyclotella* spp. AgNPs indicated the existence of carbon, suggesting the presence of an organic layer surrounding the AgNPs. This organic layer is likely formed by the biomolecules derived from the microalgae cells, indicating their potential role in the synthesis and stabilization of the AgNPs.

It is worth noting that the observed variations in the elemental percentages can be attributed to the distinct composition and abundance of biomolecules within each microalgae strain. Different microalgae species may possess varying biomolecular profiles, which could influence the composition and properties of the synthesized AgNPs. These findings align with numerous studies that have reported similar observations [27,28].

**Figure 5:**
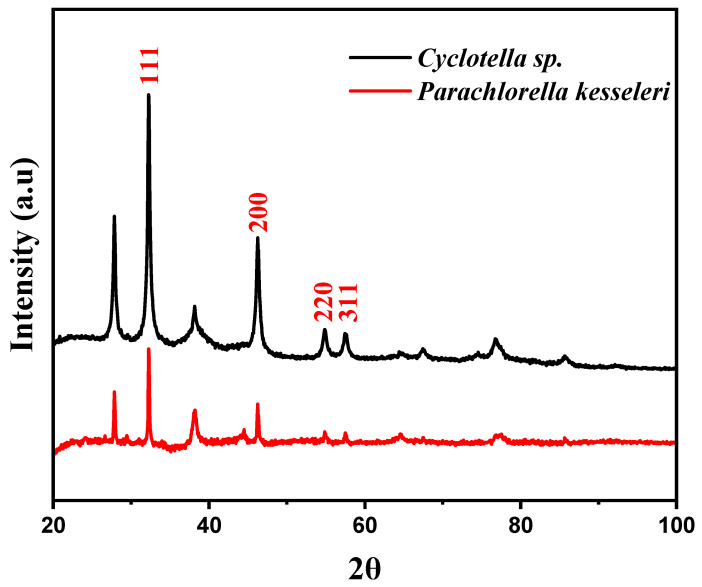
XRD profile of *P. kesseleri* and *Cyclotella* spp. AgNPs.

The crystal structure and diffraction properties of biologically generated AgNPs were examined using X-ray powder diffraction. The XRD data revealed peaks at 2θ values of 32, 46, 55, and 57.5, corresponding to the, (111), (200), (220), and (311) Bragg reflections, as shown in Figure 5. This confirms the existence of AgNPs in the sample. The peak intensity indicates a high degree of crystallinity of the AgNPs. The Debye–Scherrer formula was applied to estimate the nanoparticle size from the XRD results, yielding an average crystallite size of around 30 nm.

The diffraction peaks indicate that the AgNPs are small in size and have a face-centered cubic structure and are crystalline in nature [13,29,30]. Other peaks were also observed and have been reported in other studies that included a pertinent 2θ range in the XRD pattern. These peaks are likely generated by the presence of biological molecules within the microalgae cells [31].

**Figure 6:**
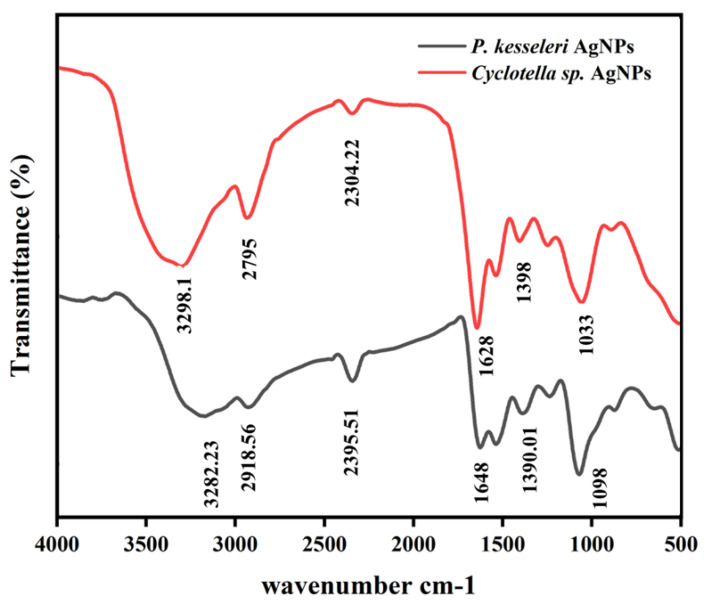
FTIR spectra of biosynthesized profile AgNPs.

The identification of possible biomolecules involved in the reduction in Ag+ ions and capping of reduced AgNPs in *P. kessleri* and *Cyclotella* spp. cells was performed by FTIR measurements (Figure 6). Spectral analysis revealed significant peaks at 3281.23, 2918.56, 2395.51, 1648, 1390.01 and 1098 cm*^−^*^1^ for *P. kessleri* AgNPs, and at 3298.1, 2795, 2304.22, 1628, 1398 and 1033 cm*^−^*^1^ for *Cyclotella* spp. AgNPs (Table S1). The peaks at 3281.23 and 3298.1 cm*^−^*^1^ correspond to the vibrational stretching frequency (O–H) of phenols and alcohols [32], while 2918.56 and 2795 cm*^−^*^1^ are characteristic of the vibrational stretching frequency N-H of secondary amines [33]. The peaks at 1648 and 1628 cm*^−^*^1^ were related to the N-H bending of amines associated with the amide bond in both peptides and proteins [33], and the peaks at 1390.01 and 1398 cm*^−^*^1^ corresponded to the C–H bending of alkanes [34]. In addition, the peaks at 1098 and 1033 cm*^−^*^1^ could be attributed to the C–N stretching vibration of the amine group [34]. On the basis of these results, it can be suggested that protein molecules may be implicated in the biological synthesis and stabilization of AgNPs produced by *Cyclotella* spp. and *P. kessleri*, which is in agreement with other studies indicating the role of proteins in the reduction in AgNPs [33]. Kashyap et al. conducted a study on *Chlorella* sp. to assess its potential for synthesizing Ag/AgCl NPs. They observed specific peaks in the FTIR spectra of the Ag-NPs at 3239.91, 2916.67, 2848.94, 1532.07, and 1462.05 cm*^−^*^1^. The peak at 3239.91 cm*^−^*^1^ indicated (O–H) stretching, while the peaks at 2916.67 cm*^−^*^1^ were associated with lipids and carbohydrates, specifically (CH2) and (CH2) stretching. Additionally, the peak at 1532.07 cm*^−^*^1^ represented (N–H) bending and (C–N) stretching, and the peak at 1462.05 cm*^−^*^1^ indicated the (C–O) group of COO− carboxylates. These functional groups suggested that proteins and lipids played significant roles in the reduction and stabilization process of the nanoparticles [16].

In a separate study, Hamida et al. utilized *Coelastrella aeroterrestrica* Strain BA_Chlo4 to synthesize AgNPs. The resulting AgNPs were subjected to FTIR analysis, revealing nine peaks at 3347.4, 2940.0, 2861.7, 1645.3, 1521.7, 1397.7, 1240.0, 1059.9, and 564.9 cm*^−^*^1^. The sharp peak at 3347.4 cm*^−^*^1^ indicated a strong broad O–H stretching group of alcohol or medium N–H stretching group of primary amines. Peaks at 2940.0 and 2861.7 cm*^−^*^1^ were related to a strong broad O–H stretching group of carboxylic acid or strong N–H stretching group of amine salts or a medium C–H stretching group of alkanes. The peak at 1645.3 cm^−1^ corresponded to medium C=N stretching of imine/oxime or strong C=C stretching of alkene or medium N–H bonding of amine, while the following peak at 1521.7 cm*^−^*^1^ represented strong N–O stretching of nitrocompound. The peak at 1397.7 cm^−1^ was associated with strong S=O stretching of sulfate chloride or medium O–H bending of carboxylic acid or alcohol. The authors reported that *Coelastrella aeroterrestrica* successfully reduced AgNO_3_ into Ag-NPs and capped their surfaces [35].

The concentration of AgNPs synthesized by microalgae *P. kesseleri* and *Cyclotella* spp. was quantitatively determined using Ultima2 Jobin Yvon ICP-AES. The results showed that the concentration of AgNPs after the acid digestion of the AgNP powder was 0.133 mg/L for *P. kesseleri* and 0.184 mg/L for *Cyclotella* spp. at the elemental symbol wavelength of Ag 328.068.

### 2.3. Antibacterial Activity of AgNPs

**Figure 7:**
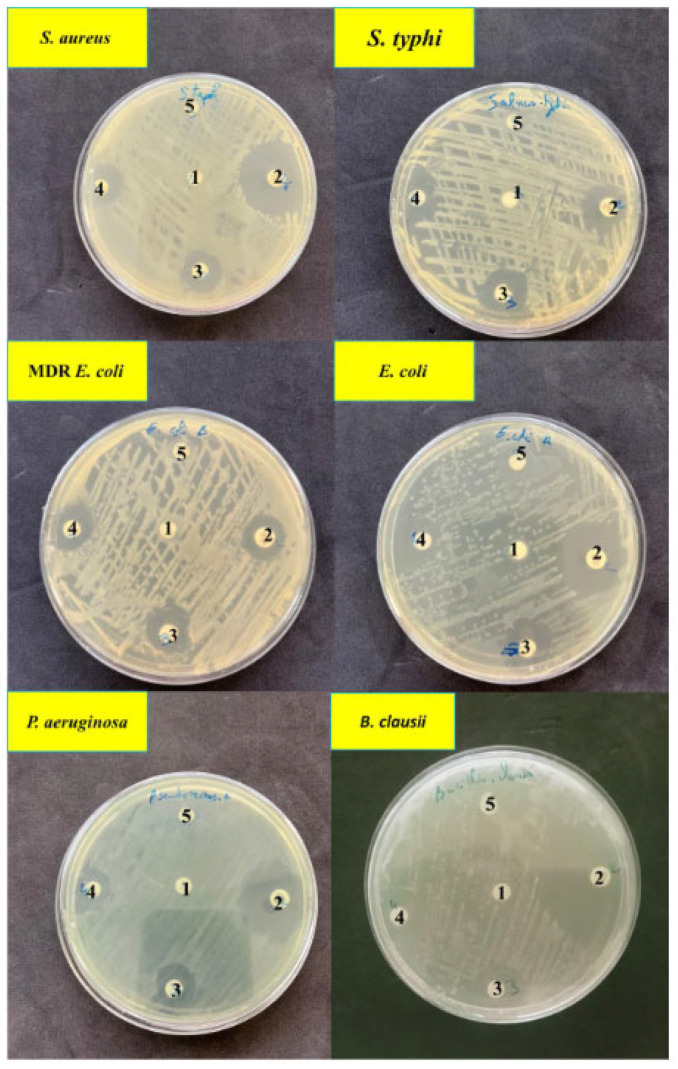
(1) Sterile virgin disk as negative control, (2) gentamicin as the standard antibiotic, (3) disk loaded with 50 µg/mL of *P. kessleri* AgNPs, (4) disk loaded with 50 µg/mL of *Cyclotella* spp. AgNPs, and (5) disk loaded with 1 mM of AgNO_3_.

The disk diffusion sensitivity method was used to evaluate the efficacy of AgNPs against four Gram-negative bacteria: MDR *E. coli*, *E. coli*, *P. aeruginosa*, and *S. typhi*, as well as two Gram-positive bacteria: *S. aureus* and *B. clausii*. The assay revealed that the nanoparticles exhibit antibacterial activity against the selected bacteria (Figure 7).

A study was conducted to compare the inhibitory effects of AgNPs synthesized from two microalgae strains and the reference antibiotic, gentamicin, on bacterial growth. The results revealed significant differences in the zone of inhibition for the six tested bacterial strains, indicating that the AgNPs exhibited higher antimicrobial activity than gentamicin for certain bacteria (Table 1). The study demonstrated that AgNPs derived from both microalgae strains possessed potent antibacterial properties against both Gram-positive and Gram-negative bacteria, with inhibition zones ranging from 13.66 ± 1.15 mm to 21.33 ± 1.15 mm. Notably, the highest antibacterial activity was observed against *B. clausii*, with a maximum inhibition zone diameter of 21.33 ± 1.15 mm.

Interestingly, the tested concentration of AgNO_3_ (1 mM) had no biocidal effect and did not generate a zone of inhibition against the bacteria tested. This suggests that either the AgNO_3_ concentration tested was too low or that the concentration of AgNO_3_ synthesizing AgNPs was crucial to provide antibacterial activity, and that silver ions alone were insufficient. Comparing the results, it is evident that the zone of inhibition against multidrug-resistant *E. coli* was slightly lower than that observed for wild *E. coli*. This indicates that the nanoparticles were effective in inhibiting the growth of both wild and drug-resistant strains of *E. coli*, although they exhibited slightly higher efficacy against the wild strain.

Furthermore, the results indicated that Gram-negative bacteria were more susceptible to the inhibitory effects of AgNPs at a concentration of 50 µg/mL compared to Gram-positive bacteria [36,37]. This difference in susceptibility is attributed to the structural characteristics of the bacterial cell walls. Gram-negative bacteria have a thinner peptidoglycan layer and an additional outer membrane composed of lipopolysaccharides, which may facilitate the easier penetration of nanoparticles and liberated ions into the cells. In contrast, Gram-positive bacteria possess a thicker peptidoglycan layer, including covalently linked teichoic and teichuronic acids, which act as a protective barrier against the invasive action of AgNPs and Ag+ ions [38].

Additionally, the presence of negatively charged lipopolysaccharide molecules on the surface of Gram-negative bacteria enhances their susceptibility to nanoparticle interactions. These molecules have a higher affinity for positively charged nanoparticles and released ions, leading to the accumulation of ions within the cells and subsequent internal damage [39].

Comparing the two types of nanoparticles, *Cyclotella* spp. AgNPs generally exhibited larger zones of inhibition against all tested bacteria compared to *P. kesseleri* AgNPs. This suggests that *Cyclotella* spp. AgNPs may possess stronger overall antimicrobial properties.

## 3. Methods and Materials

### 3.1. Collection and Cultivation of Microalgae

The microalga *Cyclotella* spp. was collected from several sites in the Agadir region of Morocco, while *Parachlorella kessleri* strain UTEX2229 was provided by the GEPEA, University of Nantes, France. The modified Bold basal medium (BBM) was used for the growth of the two strains of microalgae [40]. All glassware used in the cultivation of microalgae as well as the growth media were sterilized for 20 min at 120 °C. The two strains were grown separately at ambient temperature for 15 days, starting with an initial concentration of 5 × 10^5^ cells/mL. Cultures were performed by continuously introducing ambient air in the form of bubbles at a constant airflow of 0.5 L/min. During the lighting period, an optimal light intensity of 5000 Lux for *P. kessleri* and 800 Lux for *Cyclotella* spp. was maintained using white fluorescent lamps, with a light/dark cycle of 16/8 h. When the culture reaches the stationary phase, aliquots were taken to start new cultures.

### 3.2. Biosynthesis of Silver Nanoparticles

A 0.3 M solution of silver nitrate (AgNO_3_) was made by mixing 1.53 g of this compound with 30 mL of distilled water. Logarithmic phase cultures of *P. kessleri* were collected, centrifuged five minutes at 4500 rpm, and washed three times with distilled water to remove unwanted components. The reconstituted cells were then mixed with 100 mL of distilled water, and 0.34 mL of the AgNO_3_ mother solution were added to achieve a final concentration of 1 mM. The cell AgNO_3_ mixture was then incubated for 24 h at room temperature under light exposition. A control solution containing only *P. kessleri* without AgNO_3_ was also prepared. The pH and light intensity effect on the AgNPs formation was evaluated at four pH levels (4, 7, 8 and 10) and at different light intensities. The same synthesis protocol described for *P. kesseleri* was performed for the production of AgNPs by *Cyclotella* spp. The stability of the biosynthesized AgNPs was determined by storing the samples in the dark at room temperature for several months.

### 3.3. Characterization of AgNPs

The reaction color change was observed visually. The silver ion bioreduction was periodically checked with a UV-Visible spectrophotometer (Rayleigh UV-1800 V/VIS, Beijing, China) to identify the absorbance of the reaction. The morphology of the AgNPs was determined using a JOEL JSM-IT100 scanning electron microscope (SEM). For qualitative and quantitative elemental analysis, the system is equipped with an integrated JEOL X-ray energy dispersive spectroscopy (EDS). The structural analysis of the synthesized AgNPs was performed using a Bruker D8 Advance X-ray diffractometer, with Cu-Kα radiation (λ = 1.5406 Å), and a 2θ range of 0 < 2θ < 100. The size of the biosynthesized AgNPs was determined using the Debye–Scherrer formula (d = 0.9 λ/β cos θ) [41]. Fourier transform infrared (FTIR) spectral measurements were conducted to identify possible molecules responsible for the reduction in Ag^+^ ions and capping of bioreduced AgNPs by living microalgae cells. The IRTracer-100 (Shimadzu) was utilized to record the Fourier transform infrared spectra of AgNPs in the range of 4000–500 cm^−1^. A KBr pellet was utilized as the background. Quantitative analysis of AgNPs was performed using Ultima2 Jobin Yvon ICP-AES. The elemental concentration of silver was determined by repeatedly centrifuging the supernatant containing AgNPs that had settled in the dark at ambient temperature. The pellet was then digested with 70% nitric acid for 8 h. After filtration, the digested sample was analyzed by ICP-AES.

### 3.4. Evaluation of the Antibacterial Efficacy of AgNPs

The AgNPs antibacterial activity against six strains of bacteria, *Escherichia coli* ATCC 25922, MDR *Escherichia coli*, *Salmonella typhi*, *Pseudomonas aeruginosa* ATCC27853, *Staphylococcus aureus* ATCC29213, and *Bacillus clausii,* was studied using the Kirby–Bauer disc diffusion sensitivity test method [42]. Each bacterial strain was streaked using sterile cotton swabs onto Mueller Hinton agar. A sterile virgin disk was prepared as a control. Gentamicin was used as the standard antibiotic. Three other disks were loaded with 1 mM AgNO_3_, 50 μg/mL *P. kessleri* AgNPs, and 50 μg/mL *Cyclotella* spp. AgNPs. The disks were positioned on the agar plate and incubated for 24 h at 37 °C. The inhibition zone (IZ) was noted after 24 h of incubation. To determine the minimum inhibitory concentration (MIC) of AgNPs, broth dilution method [43] was used. The six strains of bacteria were exposed to final concentrations of 6, 12, 25, 50, and 100 μg/mL of AgNPs in freshly prepared sterile Mueller Hinton broth. Positive control tubes were not supplemented with AgNPs. A volume of 100 μL of a 0.5 McFarland suspension of each bacterial strain was inoculated into each tube, except for the negative control, and then incubated for 24 h at 37 °C. The experiment was repeated thrice for each strain, and turbidity was assessed the next day by comparing the tubes to positive and negative controls.

### 3.5. Statistical Analysis

The experiments were conducted in triplicates, and statistical analysis was performed using IBM SPSS Statistics 23. The results are presented as the mean ± standard deviation (SD). Intergroup differences were assessed through one-way analysis of variance (ANOVA), followed by a post hoc multiple comparisons test (Tukey test). Statistical significance was considered at a significance level of *p* < 0.05.

## 4. Conclusions

In this study, we successfully synthesized silver nanoparticles (AgNPs) using two green microalgae strains, *P. keseleri* and *Cyclotella* spp., resulting in AgNPs with a size range of 20 to 50 nm. The biosynthesized AgNPs exhibited similar shapes and sizes when produced by both microalgae strains. Furthermore, the remarkable antibacterial activity demonstrated by these bioactive AgNPs against six bacterial strains highlights their potential as effective antimicrobial agents in various biomedical and environmental applications.

However, it is important to acknowledge the need for further investigations regarding the toxicity and safety of these AgNPs before considering their application in the medical field. Additional studies are required to comprehensively understand the potential risks and evaluate their suitability for use in medical contexts.

The utilization of microalgae cultures for the biosynthesis of AgNPs exemplifies a sustainable and environmentally friendly method for nanoparticle synthesis. However, further research is necessary to elucidate the precise mechanism underlying the biosynthesis process and to investigate the interactions between these nanoparticles and bacteria. These studies will yield valuable insights, contributing to future research endeavors in this field and facilitating a deeper understanding as well as potential advancements in the synthesis and application of microalgae-mediated AgNPs.

## Figures and Tables

**Table 1:** Antibacterial activity of biosynthesized AgNPs by (a) *P. kesseleri* and (b) *Cyclotella* spp. (ND: Not detected).

The Tested Strains	AgNO_3_	AgNPs (a)		AgNPs (b)		Gentamycin
	IZ (mm)	IZ (mm)	MIC (µg/mL)	IZ (mm)	MIC (µg/mL)	IZ (mm)
***E. coli*** **ATCC25922**	ND	14.33 ± 0.57 b	25	17.33 ± 1.15 bc	25	16.00 ± 00 b
**MDR *E. coli***	ND	13.66 ± 0.57 a	25	15.00 ± 0.00 a	25	13.66 ± 0.57 a
***P. aeruginosa* ATCC27853**	ND	16.66 ± 0.57 b	25	15.33 ± 0.57 a	25	23.33 ± 0.57 c
***S. aureus*** **ATCC29213**	ND	13.66 ± 1.15 a	50	16.66 ± 0.57 abc	50	27.33 ± 0.57 d
***B. clausii***	ND	20.66 ± 1.52 c	50	21.33 ± 1.15 d	50	34.66 ± 0.57 e
***S. typhi***	ND	16.66 ± 1.15 b	25	18.00 ± 1.00 c	25	16.33 ± 0.57 b
**Valeur *p***		*p* = 0.00 *		*p* = 0.00 *		*p* = 0.00 *

The results are expressed as mean ± standard deviation (*n* = 3). The *p*-value or Sig (two-tailed) represents the risk of type 1 error or alpha risk. The letters a, b, c, d, and e, they are indicating homogeneous subsets. They are automatically formed in Post hoc analysis (tukey test) using one-way ANOVA in SPSS, with a significance threshold of 0.05. Different letters showed different homogeneous groups. (*) indicates a significant difference between the groups when the *p*-value is lower than 0.05.

## Data Availability

Any data or material that support the findings of this study can be made available by the corresponding author upon request.

## Supplementary Materials

The following supporting information can be downloaded at: https://www.mdpi.com/article/10.3390/ijms241310599/s1.
