# Supplementary material for: Biosynthesis and Characterization of Silver Nanoparticles Produced by Parachlorella kessleri and Cyclotella spp., and the Evaluation of Their Antibacterial Activity
Source: Int J Mol Sci. 2023 Jun 25;24(13):10599. doi: 10.3390/ijms241310599 (PMC10341437; doi:10.3390/ijms241310599)
Supplement: Supplementary file 1 [file ijms-24-10599-s001.zip › ijms-2354947-supplementary.pdf]

Table S1: FTIR shows the functional group involved in biosynthesis of silver nanoparticles using *P. kessleri* and *Cyclotella spp.*

	AgNPs using *P. kesseleri*			AgNPs using *Cyclotella spp.*		
No.	Absorption peak (cm^{-1})	Vibration Mode	Functional groups	Absorption peak (cm^{-1})	Vibration Mode	Functional groups
1	3281.23	O-H structure	Phenols	3298.1	O-H structure	Phenols
2	2918.56	N-H structure	Secondary amines	2795	N-H structure	Secondary amines
3	2395.51	NH structure	Charged amines	2304.22	NH structure	Charged amines
4	1648	NH structure	Charged amines	1628	NH structure	Charged amines
5	1390.01	C-H structure	Alkanes	1398	C-H structure	Alkanes
6	1098	C-N structure	Aliphatic amines	1033	C-N structure	Aliphatic amines
